# Gender Affirmation Surgery

**DOI:** 10.1155/2018/1768414

**Published:** 2018-07-05

**Authors:** Gennaro Selvaggi, Christopher J. Salgado, Stan Monstrey, Miroslav Djordevic

**Affiliations:** ^1^Department of Plastic Surgery, Institute of Clinical Sciences, Sahlgrenska Academy, University of Gothenburg, Sahlgrenska University Hospital, Gothenburg, Sweden; ^2^Division of Plastic Surgery, University of Miami Miller School of Medicine, Miami, FL, USA; ^3^Department of Plastic Surgery, University Hospital Gent, Gent, Belgium; ^4^Belgrade Center for Genital Reconstructive Surgery, Belgrade, Serbia; ^5^School of Medicine, University of Belgrade, Belgrade, Serbia

Gender Affirmation Surgery (GAS) is a collection of surgical procedures performed in patients presenting with diagnosis of “Gender Dysphoria” (according to the Diagnostic Statistical Manual of Mental Disorders (DSM-V), published by the American Psychiatric Association [1] (2013)) or “Transsexualism” (according to the International Classification of Diseases (ICD-10), published by the World Health Organization [2, 3] (WHO, 1992; WHO, 2007)).

Gender Dysphoria (GD) and Transsexualism (T) are both referring to the “discomfort or distress caused by the discrepancy between a person's gender identity and that person's sex assigned at birth” [4, 5] (Fisk, 1974; Knudson, De Cuypere, and Bockting, 2010). Mental health professionals are in charge of making a diagnosis; however as surgeons operating on patients we need to agree with the diagnosis prior to our interventions.

The treatment for GD may consist of mental health therapy, cross-sex hormone therapy, and different forms of surgery. More specifically, GAS refers to the whole genital, facial, and body procedures required to create a body phenotype that best represents one's own identity. The requested body phenotype is not always representing a fully masculine or feminine aspect, and this may be representative of patient satisfaction of their desired goals in transition. Many individuals, in fact, might opt to undergo only some of the surgical procedures currently available, while others opt to receive the full collection of treatments. Those who are requesting only part of the collection of procedures, for example, trans-men seeking mastectomy and not penile construction, might do so either because they represent a gender nonconforming identity or because they realize that the surgical technique(s) available will not fulfill their expectations.

Indeed, there are also individuals that, although having discrepancy between one's own gender and that assigned at birth, might experience only a minimum distress, or no distress at all, and therefore they might opt for other treatments (mental health therapy and/or hormonal therapy) but surgery, or no treatment at all.

To date, the majority of the academic centers worldwide are managing this condition according to the Standards of Care [6] as proposed by the World Professional Association of Transgender Health.

Gender Affirmation Surgery remains an area of super-specialization and is in constant development. Few surgical refinements have been published within the last few years, and follow-ups of long-term series of patients proceed slowly. Cooperation among centers represents a solution to standardize approaches and techniques and achieves a higher level of evidence. This cooperation is realized through reciprocal visiting and meetings, discussion during conferences, and live-surgery sessions in order to learn “the way he/she does it”. At the same time, multicenter studies are to be promoted, in order to collect large series of cases that would add to the evidence of a given procedure. Finally, standardized and validated methods of assessment need to be defined (Patient Reported Outcome Measures) [7, 8], in order to measure objectively, without bias, the outcomes of each treatment.

Lastly, the development of medical and surgical techniques must advance in parallel to ethical discussions on the permissibility of specific treatments, research protocols, or more innovative surgeries. In fact, the large variability of the transgender population (considering both one's experience of the gender identity and the relationship within one's sociocultural background) poses ethical questions that often go beyond medical ethics and extend to areas of sociopolitical sciences and human rights. In this issue, we highlight scientific advances made in the field of Gender Affirmation Surgery with an international collaborative approach that allows this issue to be both comprehensive and informative.

*Gennaro Selvaggi* *Christopher J. Salgado* *Stan Monstrey* *Miroslav Djordevic*
